# Muscle quality indices separately associate with joint-level power-related measures of the knee extensors in older males

**DOI:** 10.1007/s00421-022-05005-2

**Published:** 2022-07-18

**Authors:** Kosuke Hirata, Mari Ito, Yuta Nomura, Chiho Kawashima, Yuma Tsuchiya, Kosuke Ooba, Tsukasa Yoshida, Yosuke Yamada, Geoffrey A. Power, Neale A. Tillin, Ryota Akagi

**Affiliations:** 1 Faculty of Sport Sciences, Waseda University, Tokorozawa-shi, Saitama Japan; 2 Airweave Inc. Nukata-gun, Aichi, Japan; 3 Graduate School of Engineering and Science, Shibaura Institute of Technology, Saitama-shi, Saitama Japan; 4 College of Systems Engineering and Science, Shibaura Institute of Technology, Saitama-shi, Saitama Japan; 5 Section of Healthy Longevity Research, National Institute of Health and Nutrition, National Institutes of Biomedical Innovation, Health and Nutrition, Shinjuku-ku, Tokyo, Japan; 6 Department of Human Health and Nutritional Sciences, College of Biological Science, University of Guelph, Guelph, ON Canada; 7 School of Life and Health Sciences, University of Roehampton, London, UK

**Keywords:** Rate of power development, Rate of velocity development, Echo intensity, Bioelectrical impedance spectroscopy, Intracellular water, Specific muscle strength

## Abstract

**Purpose:**

The purpose of this study was to investigate associations of muscle quality indices with joint-level power-related measures in the knee extensors of thirty-two older males (65–88 years).

**Methods:**

Muscle quality indices included: echo intensity, ratio of intracellular- to total water content (ICW/TW), and specific muscle strength. Echo intensity was acquired from the rectus femoris (EI_RF_) and vastus lateralis (EI_VL_) by ultrasonography. ICW/TW was computed from electrical resistance of the right thigh obtained by bioelectrical impedance spectroscopy. Specific muscle strength was determined as the normalized maximal voluntary isometric knee extension (MVIC) torque to estimated knee extensor volume. Isotonic maximal effort knee extensions with a load set to 20% MVIC torque were performed to obtain the knee extension power-related measures (peak power, rate of power development [RPD], and rate of velocity development [RVD]). Power and RPD were normalized to MVIC.

**Results:**

There were no significant correlations between muscle quality indices except between EI_RF_ and EI_VL_ (|*r*|≤ 0.253, *P* ≥ 0.162). EI_RF_ was negatively correlated with normalized RPD and RVD (*r* ≤  − 0.361, *P* ≤ 0.050). ICW/TW was positively correlated with normalized peak power (*r* = 0.421, *P* = 0.020). Specific muscle strength was positively correlated with absolute peak power and RPD (*r* ≥ 0.452, *P* ≤ 0.012).

**Conclusion:**

Knee extension power-related measures were lower in participants with higher EI, lower ICW/TW, and lower specific muscle strength, but the muscle quality indices may be determined by independent physiological characteristics.

## Introduction

Natural adult aging is associated with a loss of muscle mass, motor units, and alters fiber type and myosin heavy-chain isoform expression (Power et al. 2013). These age-related alterations lead to decreases in muscle strength and power, with the reduction of muscle power being greater than muscle strength (McNeil et al. 2007). Importantly, muscle power is a better predictor of physical function compared with muscle strength in older adults (Bean et al. 2010; Foldvari et al. 2000). Hence, investigating the neuromuscular factors influencing muscle power in older adults may help inform research and practice aimed at maintaining independence and improving quality of life, in older adults.

Maximal power production is dependent on a variety of factors including, but not limited to muscle volume (Thom et al. 2005) and neuromuscular activity (McKinnon et al. 2017). Muscle quality may also be a determining factor of muscle power, since muscle quality is recognized to influence muscle function, especially in older adults (Goodpaster et al. 2006). There are several indices of muscle quality which can be assessed in the context of voluntary power production, including: echo intensity (EI), intracellular water (ICW) to total water (TW) ratio (ICW/TW) within muscle, and specific muscle strength. These muscle quality indices reflect multiple aspects of muscle quality. EI evaluated by B-mode ultrasonography represents brightness of ultrasonographic image, and is influenced by intramuscular fat (Akima et al. 2016) and fibrous tissue content (Pillen et al. 2009). An age-related increase in EI occurs due to fat and fibrous tissue accumulation with age (Akima et al. 2016; Pillen et al. 2009). Ratios between ICW, extracellular water, and TW content within body or limbs, estimated by bioelectrical impedance spectroscopy, is a proxy of extracellular space within muscle (Yamada et al. 2010) which increases with age (Lexell et al. 1988). Thus, a lower ICW/TW can be interpreted as fewer muscle fibers per unit of whole muscle area (Yamada et al. 2010). Specific muscle strength, determined as the force per unit muscle size (i.e., myofibular protein content) (Fukunaga et al. 1996), is also an index of muscle quality. With natural adult aging, there is an uncoupling of the muscle size/strength relationship, with greater losses in muscle strength than would be expected based on anatomical cross-sectional area (Mitchell et al. 2012), suggesting specific muscle strength decreases with age.

Although age-related maintenance of the above-listed muscle quality indices are known to optimize static muscle strength (Castro et al. 1995; Taniguchi et al. 2017; Yoshida et al. 2018), influences of muscle quality on muscle power-related measures have not been widely studied. Higher quadriceps femoris EI (reflecting lower muscle quality) has been associated with lower knee extension power and counter-movement jump performance in older adults (Wilhelm et al. 2014). Additionally, a higher ratio of extracellular water to ICW of the leg (reflecting lower muscle quality) has been correlated with lower leg extension power during vertical jump (Yamada et al. 2017). However, the aforementioned study investigated muscle power during multi-joint performance, which may have limited the associations observed between muscle quality and power, because multi-joint performance is influenced by multiple muscle groups and movement coordination. Investigating muscle quality and power at the single-joint level may provide a clearer understanding of the association between muscle quality and power. Additionally, compared to peak power, rate of power development (RPD) and rate of velocity development (RVD) may be more sensitive indicators of age-related deficits in skeletal muscle functional capacity (Thompson et al. 2014; Van Roie et al. 2018). Hence, it would be important to explore how multiple muscle quality indices relate to time-dependent measurements of single-joint-level power. In the present study, we defined single-joint-level power as instantaneous peak power of single-joint movement, and single-joint-level power was calculated as a product of single-joint torque and single-joint angular velocity.

Therefore, the purpose of this study was to investigate associations of the muscle quality indices (EI, ICW/TW and specific muscle strength) with single-joint-level power-related measures (peak power, rate of power development [RPD] and RVD) in older men. We hypothesized that joint-level power-related measures would be lower in participants with higher EI, lower ICW/TW, or lower specific muscle strength.

## Materials and methods

### Participants

To calculate the sample size for correlation analysis, a priori power analysis was performed using G*Power statistical power analysis software (G*Power 3.1.7; Kiel University, Germany). A type 1 error and a statistical power were set at 0.05 and 0.80, respectively. Based on the previous studies (Wilhelm et al. 2014; Yamada et al. 2017), we assumed an effect size of 0.50. The critical sample size was calculated to be 26, and we recruited 32 older males, ensuring an adequate sample size. Participant physical characteristics are reported in (Table 1). No participants reported any orthopedic or neurological disorders, muscle soreness, or fatigue at the time of testing. All participants were informed of the purpose and risks of this study and were required to give written informed consent. The experimental procedure was approved by the ethics committee of the Shibaura Institute of Technology. This study was performed in accordance with the Declaration of Helsinki.

**Table 1:** Physical characteristics, routine physical activities, muscle quality, and knee extension power-related measures of the participants

	Mean	SD	Median	Inter-quartile range	Min	Max	95% CI	
							Lower limit	Upper limit
Physical characteristics								
Age (year)	74	6	73	7	65	88	72	76
Height (cm)	166	7	165	10	150	177	164	169
Weight (kg)	66	10	64	11	48	86	62	69
BMI (kg/m^2^)	24	3	23	5	19	30	23	25
Thigh length (cm)	36	2	36	3	32	41	36	37
MT_anterior thigh_ (mm)	42	4	43	6	32	51	41	44
MV_QF_ (cm^3^)	1159	153	1174	187	860	1518	1104	1214
Routine physical activity								
Light (min/day)	452	93	457	110	216	696	419	486
Moderate (min/day)	76	35	71	47	15	146	63	89
Vigorous (min/day)	1.9	3.3	0.9	1.2	0.1	16	0.8	3.1
MVPA (min/day)	78	37	73	48	16	159	65	91
Muscle quality								
EI_RF_ (a.u.)	91	7	91	8	79	108	89	94
EI_VL_ (a.u.)	91	5	92	7	76	103	89	93
ICW/TW	0.688	0.039	0.687	0.036	0.556	0.763	0.674	0.702
Specific muscle strength (Nm/cm^3^)	0.095	0.019	0.094	0.025	0.047	0.154	0.088	0.102
Muscle strength								
MVIC torque (Nm)	110	25	110	26	50	191	101	119
P_peak_ (W)	270	63	271	89	99	376	247	292
RPD_0–50_ (W/s)	2271	789	2258	921	589	4169	1987	2555
Normalized P_peak_ (%MVIC‧rad/s)	245	39	250	60	175	319	232	259
Normalized RPD_0–50_ (%MVIC‧rad/s^2^)	2068	637	2061	508	611	3507	1838	2298
RVD_0–50_ (rad/s^2^)	39	10	38	15	12	57	35	43

*SD* standard deviation, *Min* minimum value, *Max* maximum value, *SE* standard error, *CI* confidence interval, *BMI* body mass index, *MT* muscle thickness, *QF* quadriceps femoris, *MV* muscle volume, *MVPA* moderate- to vigorous-intensity physical activity, *EI* echo intensity, *RF* rectus femoris, *VL* vastus lateralis, *ICW/TW* intracellular- to total water ratio, *MVIC* maximal voluntary isometric contraction, *P*_*peak*_ peak power, *RPD* rate of power development, *RVD* rate of velocity development

### Procedures and data recording

During all testing sessions, the room temperature was set to 23 ℃. An ICW/TW measurement was conducted using a bioelectrical impedance analyzer (SFB7; ImpediMed, Australia). Ultrasonic measurements were performed to evaluate anterior thigh thickness and EI of the rectus femoris (EI_RF_) and vastus lateralis (EI_VL_). Then, the participants sat on a dynamometer seat (CON-TREX MJ; Physiomed, Germany) with hip flexed at 80° and knee flexed at 90°, and they were secured to the seat and lever arm of the dynamometer with non-elastic seat belt and strap. Center of rotations of the knee joint and lever arm were aligned visually. The participants were asked to perform maximal voluntary isometric contractions (MVICs) of the knee extensors twice after several warm-up submaximal muscle contractions. In any instance when 2 values of MVIC torque were different by more than 10% of the highest value, additional trials were conducted until the difference between the highest and second highest values was less than 10% of the highest value (1 min of rest was allowed between contractions). The maximum value of MVIC torque was used for setting the isotonic contraction load (20% MVIC torque) and further analyses. For knee extension power measurement, 10 isotonic knee extensions were performed (specific details outlined below). When performing maximal voluntary contractions (MVIC and power measurements), strong verbal encouragement and visual feedback of joint torque were provided for the participants. The torque and lever-arm angle signals were stored on a personal computer using LabChart software (ver.8; ADInstruments, Australia) through an A/D converter (PowerLab 16/35; ADInstruments). The signals were synchronized, digitized at 2 kHz, and were filtered with 500 Hz low-pass filters.

### EI

Transversal ultrasonic images were acquired from the rectus femoris (RF) and vastus lateralis (VL) three times using an ultrasonic apparatus (ACUSON S3000; Siemens Medical Solutions, USA) coupled with a linear transducer array (9 L4 Transducer, 4–9 MHz, 4 cm footprint, Siemens Medical Solutions). The participants sat on the dynamometer seat with hip flexed at 80° and knee flexed at 90°, and they were asked to relax completely. The measurement sites of EI_RF_ and EI_VL_ were at 50% of thigh length from the greater trochanter to the articular cleft between the lateral femoral condyle and lateral tibial condyle in the longitudinal direction, and at the center of each muscle width in the transverse direction. The ultrasonography settings remained constant for all participants; specifically, depth, focus, and gain of the ultrasonic image were 6 cm, at top of the image, and 90 dB, respectively. The depth of 6 cm was selected because we expected any muscle thickness of the participants did not exceed 6 cm. Prior to the EI measurement, the participants were resting in the seated position for 10 min to eliminate potential effects of fluid shift. For each ultrasonic image, EI was computed as the mean value of gray scale expressed in arbitrary units as a value between 0 (black) and 255 (white) within a region of interest (ROI) using image processing software (ImageJ; National Institutes of Health, Bethesda, MD). The ROI covered as much muscle as possible without aponeuroses, fascia, or nontarget muscles. The EI values measured in each of the three separate images collected, were averaged for each muscle (RF and VL), and the mean EI values were used as representative values for further analyses. The analysis of EI was conducted by the same investigator (RA).

### ICW/TW

To calculate ICW/TW, biological impedance spectroscopy measurement was conducted. Measurement details are described elsewhere (Yamada et al. 2010). Briefly, the participants were asked to refrain from bathing, eating or drinking for 1 h and from strenuous exercise for 24 h preceding the experiments for data accuracy. Also, prior to the measurement, the participants were asked to lay prone on a stretching mat for 10 min to eliminate the effect of immediate whole-body fluid shift. Current injection electrodes (20 mm × 20 mm, Red Dot; 3 M, US) were placed on the dorsal surfaces of the right hand and foot. Sensing electrodes were attached over the greater trochanter and the lateral aspect of the knee joint space between the lateral femoral condyle and the lateral tibial condyle of the right lower limb. The resistances of the extracellular water (*R*_ECW_) and TW (*R*_TW_) compartments for the right thigh were measured three times using the SFB7 (ImpediMed, Australia). Mean values of each resistance were used for further analyses. The resistance of the ICW compartment (*R*_ICW_) was computed by the following formula: *R*_ICW_ = 1/([1/*R*_TW_] – [1/*R*_ECW_]). ICW and ECW in the right thigh were calculated using the following equations: ICW = *ρ*_ICW_ × *L*^2^/*R*_ICW_ and ECW = *ρ*_ECW_ × *L*^2^/*R*_ECW_, where *ρ*_ICW_ (273.9 Ωcm) and *ρ*_ECW_ (47 Ωcm) represent factors for intracellular and extracellular resistivities, respectively, and *L* is the right thigh length. TW was calculated as the sum of ICW and ECW, and ICW/TW was computed by dividing ICW by TW.

### Specific muscle strength

In the present study, specific muscle strength was defined as the MVIC torque per muscle volume. MVIC torque measurement was described earlier. Muscle volume was estimated from the anterior thigh thickness and thigh length using an equation provided by a previous study (Nakatani et al. 2016). For muscle thickness measurement, transversal ultrasonic images were obtained at 50% of thigh length in longitudinal direction, and forefront of the thigh in transverse direction using the ultrasonic apparatus. The participants were asked to stand upright with their feet shoulder-width apart. Muscle thickness was evaluated as a distance between upper surface of RF and that of the femur.

### Knee extension power-related measures

**Fig. 1:**
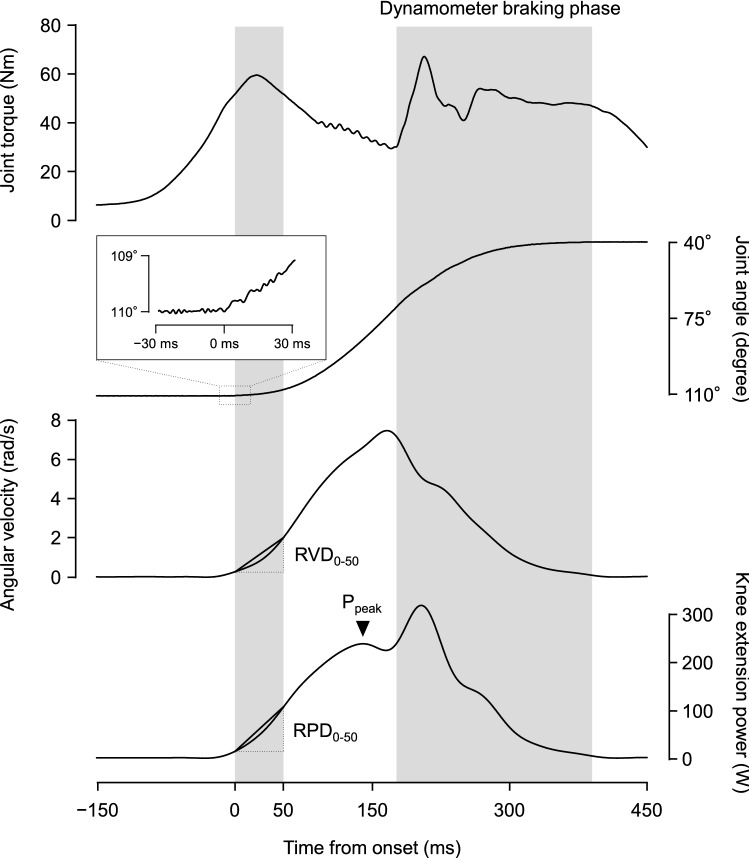
Representative waveforms during isotonic knee extension. Joint torque rises before the onset (start of lever-arm movement) to overcome the threshold (20% MVIC torque). RVD_0–50_: rate of velocity development over the interval of 0–50 ms from the onset, P_peak_: peak power, RPD_0–50_: rate of power development over the interval of 0–50 ms from the onset

Ten maximal effort isotonic knee extensions were performed with a load set to 20% MVIC torque with a 2-s rest period between contractions, using the multi-joint dynamometer (CON-TREX MJ; Physiomed, Germany). Influence of muscle fatigue or insufficient warm-up on knee extension power measurement was considered to be negligible, because no systematic increase or decrease in knee extension power-related measures during the trial were observed. The hip joint was set at 80° of flexion and the knee joint range of motion was 70° (110–40° knee flexion). Knee extension power waveform was introduced by multiplying joint torque by angular velocity. Angular velocity waveform was computed by differentiating joint angle signal. Knee extension power and angular velocity waveforms were filtered with 20-Hz low-pass filter. Knee extension power-related measures were determined as follows (Fig. 1). Peak instantaneous power was determined as the maximum value of knee extension power observed before the braking phase of the dynamometer. For calculation of the time-dependent variables (RPD and RVD), onset of power and angular velocity was defined as the last trough before joint angle deflection above the 3 times of standard deviation of the baseline noise of the time-angle curve. RPD_0–50_ and RVD_0–50_ were determined as the slopes of time-knee extension power and time-angular velocity curves over the interval of 0–50 ms, respectively. Normalized values of peak power and RPD_0–50_ were calculated by dividing absolute peak power and RPD_0–50_ by MVIC torque, respectively. The mean values of the knee extension power-related measures were obtained using the trials containing the highest three peak power, and these mean values were used for further analyses.

### Routine physical activities

Daily physical activity was assessed using an activity monitor (Active style Pro HJA-750C; Omron Health Care, Japan) for 11 days after the first day of muscle quality and power assessments. The participants were asked to wear the activity monitor except when bathing or sleeping and continue their routine daily activities. We analyzed the data except when the participant forgot to wear the activity monitor for more than 2 h, based on the participants’ declaration about wearing time. The mean wearing days was 10.7, ranging from 9 to 11. Based on metabolic equivalents (METs) measured by the activity monitor in 0.1 METs increments, physical activity was divided into three levels: light (1.1–2.9 METs), moderate (3.0–5.9 METs), and vigorous intensities (≥ 6.0 METs) (Gando et al. 2010). Then, time spent in moderate- to vigorous-intensity physical activities (MVPA) was calculated (Wu et al. 2017).

### Statistical analyses

Shapiro–Wilk test revealed that several dependent variables were not normally distributed. Hence, all data were analyzed following a rank transformation. Because muscle quality and muscle power are related with age and/or routine physical activities (Ramsey et al. 2021; Yamada et al. 2017), partial correlation analyses were performed to test associations among muscle quality indices (EIs, ICW/TW, and specific muscle strength) and knee extension power-related measures (peak power, RPD_0–50_, and RVD_0–50_), controlling for age and MVPA. Statistical tests were performed using SPSS Statistics 25 (IBM Japan, Japan). The partial correlation coefficient *r* was regarded as the effect size. We considered effect sizes *r* ≥ 0.5 as large effects, ≥ 0.3 as medium effects, and ≥ 0.1 as small effects (Cohen 1988). P ≤ 0.05 is considered to be statistically significant.

## Results

### Partial correlations between muscle quality indices

(Table 2) shows partial correlation coefficients between EIs, ICW/TW and specific muscle strength with controlling for age and routine daily activities. A correlation was confirmed only between EI_RF_ and EI_VL_ (*r* = 0.475, *P* = 0.006).

**Table 2:** Partial correlation coefficients between muscle quality indices controlled for age and routine daily activities

	EI_RF_	EI_VL_	ICW/TW	Specific muscle strength
EI_RF_				
* r*	−	0.475	− 0.253	− 0.018
* P*	( −)	(0.006)	(0.162)	(0.922)
EI_VL_				
* r*		−	− 0.017	0.078
* P*		( −)	(0.926)	(0.673)
ICW/TW				
* r*			−	0.196
* P*			( −)	(0.282)
Specific muscle strength				
* r*				−
* P*				( −)

*EI* echo intensity, *RF* rectus femoris, *VL* vastus lateralis, *ICW/TW* ratio of intracellular- to total water

### Partial correlations of muscle quality indices with knee extension power-related measures

**Fig. 2:**
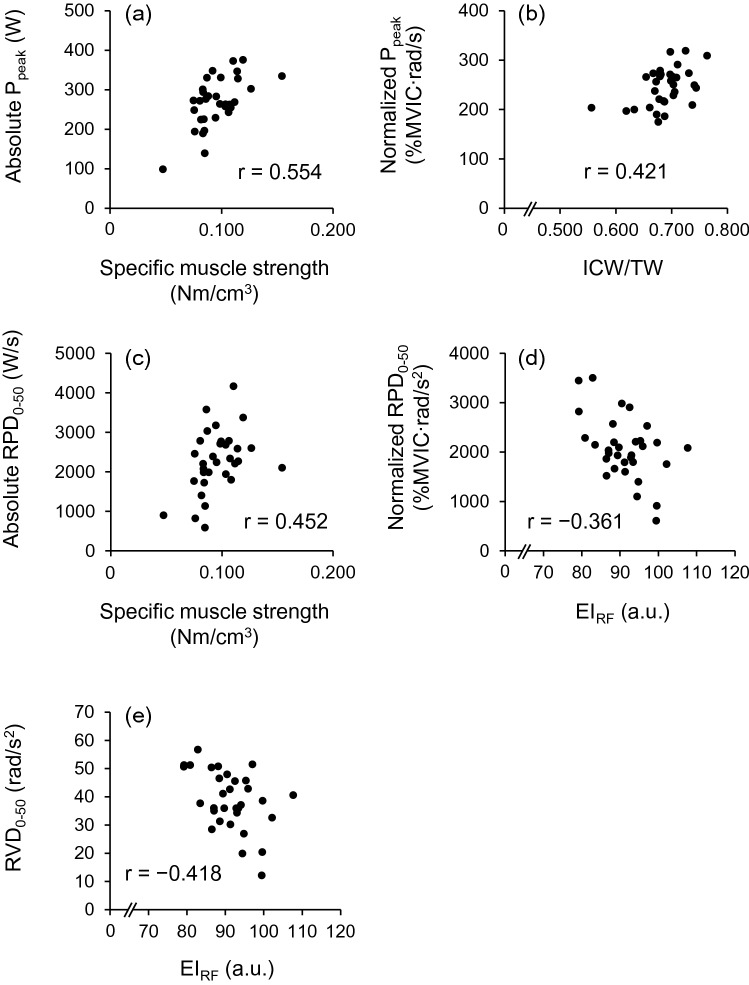
Simple correlation scatter plots between **a** specific muscle strength vs. absolute knee extension peak power (P_peak_), **b** ratio of intracellular- to total water (ICW/TW) vs. normalized P_peak_, **c** specific muscle strength vs. absolute rate of knee extension power development over the interval of 0–50 ms (RPD_0–50_), **d** echo intensity of the rectus femoris (EI_RF_) vs. normalized RPD_0–50_, and **e** EI_RF_ vs. rate of knee extension angular velocity development over the interval of 0–50 ms (RVD_0–50_). Scatter plots are shown for those with significant partial correlation. Partial correlation coefficients controlled for age and routine daily activities are included in each plot

Table 3 shows the partial correlations (controlled for age and MVPA) of muscle quality indices with knee extension power-related measures. EI_RF_ negatively correlated with nRPD_0–50_ and RVD_0–50_ (*r* ≤  − 0.361, *P* ≤ 0.050). There were no significant correlations between EI_VL_ and knee extension power-related measures (|*r*|≤ 0.331, *P* ≥ 0.074). ICW/TW positively correlated with normalized peak power (*r* = 0.421, *P* = 0.020). For specific muscle strength, positive correlations with absolute peak power (*r* = 0.554, *P* = 0.001) and RPD_0–50_ (*r* = 0.452, *P* = 0.012) were observed. Scatter plots between muscle quality indices and knee extension power-related measures are shown for those with significant partial correlation (Fig. 2).

**Table 3:** Partial correlation coefficients of muscle quality indices with knee extension power-related measures controlled for age and routine daily activities

	EI_RF_	EI_VL_	ICW/TW	Specific muscle strength
Absolute peak power				
* r*	0.230	0.331	0.259	**0.554**
* P*	(0.221)	(0.074)	(0.168)	(0.001)
Absolute RPD_0–50_				
* r*	− 0.207	0.006	0.250	**0.452**
* P*	(0.272)	(0.973)	(0.183)	(0.012)
Normalized peak power				
* r*	0.011	0.262	**0.421**	− 0.006
* P*	(0.952)	(0.162)	(0.020)	(0.975)
Normalized RPD_0–50_				
* r*	**− 0.361**	− 0.181	0.316	0.104
* P*	(0.050)	(0.338)	(0.089)	(0.586)
RVD_0–50_				
* r*	** − 0.418**	− 0.206	0.237	0.166
* P*	(0.022)	(0.274)	(0.207)	(0.380)

Significant correlation (*P* ≤ 0.05) are in bold

*EI* echo intensity, *RF* rectus femoris, *VL* vastus lateralis, *ICW/TW* ratio of intracellular- to total water, *RPD* rate of power development, *RVD* rate of velocity development

## Discussion

We aimed to investigate the associations of muscle quality indices with knee extension power-related measures. In line with our hypothesis, the correlation analyses elucidated that knee extension power-related measures were lower in participants with higher EI, lower ICW/TW, or lower specific muscle strength. There were no relationships between muscle quality indices except between EI_RF_ and EI_VL_, and each muscle quality index correlated with different knee extension power-related measures.

EI_RF_ negatively correlated with normalized RPD_0–50_ and RVD_0–50_ (Table 3). For RVD, the present result is in line with a previous study (Mota et al. 2018) reporting that lower EI of the plantar flexors associated with higher plantar flexion RVD. Regarding RPD, to the best of our knowledge, this is the first study to report the association with EI. Adipose tissue and fibrous tissue content within skeletal muscle increases with age (Csapo et al. 2014; Kent-Braun et al. 2000), and these increments relate to high EI (Akima et al. 2016; Pillen et al. 2009). Also, the age-related decrease in muscle power-related measures is well known. These suggest that age can be confounding factor for the association of EI and power-related measures. However, even when adjusting for age effects, significant associations were evident in the present study (Table 3). Unlike the normalized RPD_0–50_, EI did not correlate with absolute RPD_0–50_ (Table 3). Absolute RPD is influenced by absolute static muscle strength and its influential factors, e.g., muscle size and neural activation during MVIC. Indeed, absolute RPD_0–50_ significantly correlated with MVIC torque (*r* = 0.528 and *P* = 0.002). On the other hand, there was no correlation between EI and MVIC torque (EI_RF_: *r* = 0.131 and *P* = 0.476, EI_VL_: *r* = 0.125 and *P* = 0.494). Because EI is an index of muscle composition but not muscle size or neural factors, it would be reasonable that EI did not correlate with MVIC torque and also absolute RPD_0–50_. Because the normalized RPD is less influenced by muscle size and neural activation due to the normalization by MVIC torque, the normalized RPD would be more sensitive to muscle quality index compared with the absolute RPD. This may be the reason why EI_RF_ significantly correlated with normalized RPD_0–50_ but not absolute RPD_0–50_. Significant correlations of EI with normalized RPD_0–50_ and RVD_0–50_ imply that EI would associate with intrinsic ability to quickly increase power. One possible mechanism of the association of EI_RF_ with normalized RPD_0–50_ and RVD_0–50_ is pennation angle. A large pennation angle can positively affect muscle fiber rotation leading to greater angular velocity (Wakahara et al. 2013) and muscle power output (Akagi et al. 2020). Additionally, a larger pennation angle is related to lower EI (Ryan et al. 2015). These imply that EI may correspond to pennation angle, and therefore EI might reflect RPD and RVD. However, we note this explanation is speculative and requires further investigations to determine the underlying mechanisms. The associations of EI with normalized RPD_0–50_ and RVD_0–50_ were observed only for RF not VL. Of the quadricep muscle group, the RF contains the highest percentage of type II fibers (Johnson et al. 1973), and muscle atrophy preferentially occurs in type II fibers with age (Lexell 1995; Nilwik et al. 2013). Additionally, percentage of cross-sectional area of type II fiber contributes to rapid force production (Methenitis et al. 2019). Further, onset of muscle activation of RF in knee extension is the earliest among the quadriceps femoris (Stensdotter et al. 2003). Therefore, it is reasonable to think that not EI_VL_ but EI_RF_ influences time-dependent variables especially for early phase (RPD_0–50_ and RVD_0–50_) in older adults.

EI did not correlate with absolute or normalized peak power in the present study (Table 3). Contrary to this, Olmos et al. (2019) reported that EI of the gastrocnemii significantly negatively correlated with peak power of isotonic plantar flexion (*r* =  − 0.387, *n* = 28). Olmos et al. (2019) adopted the highest value calculated from isotonic contractions with various intensities as the individual's peak power. Also, correlation analysis was conducted for a pooled group of middle-aged and older adults without controlling for age effect in the previous study. Thus, age may have been a co-variate driving the correlation between EI and power observed by Olmos et al. (2019). Wilhelm et al. (2014) also showed a significant negative correlation between EI of the quadriceps femoris and isotonic knee extension peak power with 60% of 1 repetition maximum load. However, the participants of the previous study of Wilhelm et al. (2014) (age: 66.1 ± 4.5 years, height: 1.75 ± 0.06 m, Weight: 80.2 ± 11.0 kg, MVIC torque: 211.0 ± 50.0 Nm) were relatively younger and had higher muscle strength compared with the participants in the present study (see Table 1), which may partly explain the discrepancies in observations between Wilhelm et al. and the present study. Furthermore, difference in isotonic load between the study of Wilhelm et al. and this study (20% MVIC) may be the other reason for the discrepancy. These possibilities are speculative and reasons for the discrepancies remain unclear.

ICW/TW positively correlated with normalized peak power (Table 3). Similarly to this, previous studies (Rush et al. 2021; Yamada et al. 2017) reported that maximum jump power was correlated with an index (extracellular water per ICW) which is similar to ICW/TW. ICW/TW clearly decreases with age (Yamada et al. 2010), which means extracellular space spreads with age. Since thickening extracellular matrix is suggested to impair lateral force transmission (Zhang and Gao 2014), this can be a reason for the association of ICW/TW with peak power. ICW/TW did not correlate with RPD or RVD unlike EI. ICW/TW obtained by segmental bioelectrical impedance spectroscopy is a measure of qualities in entire thigh muscles including the knee flexors and adductor muscles but not individual muscles nor the knee extensors. Considering that RPD and RVD were correlated with EI_RF_ but not EI_VL_, RPD and RVD would be sensitive to muscle quality of individual muscle rather than that of muscle group. Thus, ICW/TW may be hard to associate with the variables affected by individual muscles like RPD and RVD.

Absolute peak power and RPD_0–50_ were correlated with specific muscle strength (Table 3). Specific muscle strength is influenced by several factors, such as neuromuscular activity level, fiber type composition, and muscle architecture (Folland and Williams 2007). These factors are also considered to influence muscle power output (McNeil et al. 2007; Power et al. 2013). As mentioned earlier, EI could relate to muscle architecture (i.e., pennation angle; Ryan et al. 2015). Additionally, an age-related decline in ICW/TW is reported to associate with age-related slowing of twitch contractile properties (e.g., a prolongation of twitch-contraction time), and possibly with changes in fiber type composition (Hirata et al. 2022). If muscle architecture and/or fiber type composition had large impacts on absolute peak power and RPD_0–50_, EI and/or ICW/TW could associate with absolute peak power and RPD_0–50_. However, as we show, no significant correlations were observed (Table 3). These findings of the present and previous studies imply that the association of specific muscle strength with absolute peak power and RPD_0–50_ may be due to factors associated with neuromuscular voluntary activation rather than muscle architecture and fiber type composition. Specific muscle strength did not correlate with normalized peak power or RPD_0–50_ (Table 3), this may be because knee extension power-related measures were normalized by MVIC torque. Absolute peak power and RPD_0–50_ were strongly associated with MVIC torque (absolute peak power: *r* = 0.702 and *P* < 0.001, absolute RPD_0–50_: *r* = 0.528 and *P* = 0.002). Since a previous study suggested that peak muscle power output is achieved at slower angular velocities for older adults than younger counterparts owing to an age-related slowing of muscle contraction velocity (Dalton et al. 2010), muscle power output may be more closely related with MVIC torque especially for older adults. Specific muscle strength also strongly correlated with MVIC torque (*r* = 0.676, *P* < 0.001); this correlation is not surprising because MVIC torque is dependent on not only muscle volume but also the influential factors of specific muscle strength. These correlations of MVIC torque with absolute peak power, RPD_0–50_, and specific muscle strength suggest that the normalization of absolute knee extension power-related measures by MVIC torque might cancel out the influence of common determinant factors of specific muscle strength and muscle power output, such as neuromuscular activity level, fiber type composition, and muscle architecture. Normalized peak power or RPD_0–50_ should be closely associated to an individual’s force–length and force–velocity relationships. On the other hand, since specific muscle strength was measured using static muscle contraction at a single-joint angle (i.e., hip flexed at 80° and knee flexed at 90°) in this study, specific muscle strength may be less sensitive to the individual difference of those relationships. Hence, normalized peak power or RPD_0–50_ was not clearly associated with specific muscle strength in this study.

Muscle quality indices did not correlate with each other except between EI_RF_ and EI_VL_ (Table 2). These results were the same, even when Spearman rank-order correlation analyses were performed without controlling for age or routine physical activities. A previous study (Taniguchi et al. 2017) also reported that there was negligible correlation between EI_RF_ and extracellular water to ICW ratio, which is similar index to ICW/TW (*r* = 0.190). These indicate that each muscle quality index provides an independent characteristic of muscle physiology. Further, each muscle quality index correlated with different knee extension power-related measures in the present study (Table 3). One possible explanation for this is that the indices of muscle quality investigated in the present study evaluate aspects of muscle quality at different levels. EI, ICW/TW, and specific muscle strength represent the quality in individual muscle level, segment level, and muscle group level, respectively. Also, influential factors of EI, ICW/TW, and specific muscle strength are not same as each other. As mentioned earlier, EI, ICW/TW, and specific muscle strength is influenced by fat and connective tissue accumulation (Akima et al. 2016; Pillen et al. 2009), extracellular space within muscle (Yamada et al. 2010), and neuromuscular activity level (Folland and Williams 2007), respectively. Hence, it may be important to understand the characteristics of each muscle quality index when considering the association between physical function and muscle quality. From the present results, absolute and normalized peak knee extension power were correlated with specific muscle strength and ICW/TW, respectively (Table 3). Muscle power is suggested as a better predictor of physical function compared with muscle strength in older adults (Bean et al. 2010; Foldvari et al. 2000). Hence, specific muscle strength and ICW/TW could help to know one’s present physical functional ability. RPD and RVD were correlated with EI_RF_ and specific muscle strength (Table 3). Since decrements of RPD and RVD are suggested to be more sensitive to age-related change in muscle function than peak power (Thompson et al. 2014; Van Roie et al. 2018), EI_RF_ and specific muscle strength may be useful for early detection of age-associated functional deficit. Because specific muscle strength correlated with both peak power and RPD, specific muscle strength might be a good indicator to possibly evaluate the present and future functional abilities of older adults.

There are several limitations in the present study. First, we assessed specific muscle strength using estimated muscle volume but not actually measured one. The estimation formula of muscle volume of the quadriceps femoris was validated with no systematic error, and was established by data of Japanese middle-aged and older adults (Nakatani et al. 2016). Hence, highly accurate estimation of muscle volume in the present study was expected. Second, EI was evaluated from the ultrasonographic image obtained by a 4-cm probe at a single location of each muscle, and was not acquired from all muscles of the knee extensor. The EI value varies among muscles and between sizes, shapes and locations of the region of interest (ROI) (Caresio et al. 2015). In this study, although ROI was set as large as possible, influences of the above-mentioned factors on EI or the association of EI with knee extension power-related measures are unknown. Third, although we discussed the potential association of muscle quality indices with knee extension power-related measures (e.g., neuromuscular activity level, intramuscular composition, muscle architecture, and fiber type composition), these variables were not assessed. If these additional measures would be assessed, further insight into the association between muscle quality and power-related measures might be possible. Lastly, this study recruited only males but not females. Because sex-related difference in change of muscle power-related measures with aging (Van Roie et al. 2018) and that in muscle quality (Potvin and Bent 1997) were suggested, there might be differences in the association of muscle quality with muscle power-related measures. Hence, it may be necessary to investigate whether the influence of muscle quality indices on knee extension power-related measures is sex dependent.

## Conclusions

The purpose of the present study was to investigate associations of the muscle quality indices with knee extension power-related measures in older men. EI_RF_ negatively correlated with RPD_0–50_ normalized by MVIC torque and RVD_0–50_. ICW/TW positively correlated with peak power normalized by MVIC torque. Specific muscle strength positively correlated with absolute peak power and RPD_0–50_. Additionally, muscle quality indices did not correlate with each other. The present results suggest that each muscle quality index is associated with an aspect of knee extension power generating capacity, but the indices are determined by independent physiological characteristics. To understand the association of muscle quality with joint-level power-related measures or physical function, it is important to comprehensively evaluate the muscle quality from multiple indices, while considering the difference in each muscle quality index.

## Abbreviations

EI: Echo intensity
ICW: Intracellular water
ICW/TW: Intracellular- to total water ratio
METs: Metabolic equivalents
MVIC: Maximal voluntary isometric contraction
MVPA: Moderate- to vigorous-intensity physical activities
RF: Rectus femoris
ROI: Region of interest
RPD: Rate of power development
RVD: Rate of velocity development
TW: Total water
VL: Vastus lateralis
